# Investments and costs of oral health care for Family Health Care

**DOI:** 10.1590/S1518-8787.2016050005771

**Published:** 2016-07-12

**Authors:** Márcia Stefânia Ribeiro Macêdo, Sônia Cristina Lima Chaves, Antônio Luis de Carvalho Fernandes

**Affiliations:** I Secretaria Municipal de Saúde de Salvador. Salvador, BA, Brasil; II Programa de Pós-Graduação em Saúde Coletiva. Instituto de Saúde Coletiva. Universidade Federal da Bahia. Salvador, BA, Brasil

**Keywords:** Dental Health Services, economics, Family Health Strategy, Costs and Cost Analysis, Investments, economics

## Abstract

**OBJECTIVE:**

To estimate the investments to implement and operational costs of a type I Oral Health Care Team in the Family Health Care Strategy.

**METHODS:**

This is an economic assessment study, for analyzing the investments and operational costs of an oral health care team in the city of Salvador, BA, Northeastern Brazil. The amount worth of investments for its implementation was obtained by summing up the investments in civil projects and shared facilities, in equipments, furniture, and instruments. Regarding the operational costs, the 2009-2012 time series was analyzed and the month of December 2012 was adopted for assessing the monetary values in effect. The costs were classified as direct variable costs (consumables) and direct fixed costs (salaries, maintenance, equipment depreciation, instruments, furniture, and facilities), besides the indirect fixed costs (cleaning, security, energy, and water). The Ministry of Health’s share in funding was also calculated, and the factors that influence cost behavior were described.

**RESULTS:**

The investment to implement a type I Oral Health Care Team was R$29,864.00 (US$15,236.76). The operational costs of a type I Oral Health Care Team were around R$95,434.00 (US$48,690.82) a year. The Ministry of Health’s financial incentives for investments accounted for 41.8% of the implementation investments, whereas the municipality contributed with a 59.2% share of the total. Regarding operational costs, the Ministry of Health contributed with 33.1% of the total, whereas the municipality, with 66.9%. Concerning the operational costs, the element of heaviest weight was salaries, which accounted for 84.7%.

**CONCLUSIONS:**

Problems with the regularity in the supply of inputs and maintenance of equipment greatly influence the composition of costs, besides reducing the supply of services to the target population, which results in the service probably being inefficient. States are suggested to partake in funding, especially to cover the team’s operational cost.

## RESUMO

**OBJETIVO:**

Estimar os investimentos para implantação e os custos operacionais de uma Equipe de Saúde Bucal modalidade tipo I na Estratégia Saúde da Família.

**MÉTODOS:**

Estudo de avaliação econômica, tipo análise de investimentos e custos operacionais de uma equipe de saúde bucal no município de Salvador, Bahia, Brasil. O cálculo dos investimentos para implantação foi obtido pela soma dos investimentos em obras civis e instalações em rateio, equipamentos, móveis e instrumentais. Para os custos operacionais, foi analisada a série histórica de 2009 a 2012 e adotou-se o mês de dezembro de 2012 para levantamento dos valores monetários vigentes. Os custos foram classificados em custos diretos variáveis (materiais de consumo) e custos fixos diretos (salários, manutenção, depreciação do capital fixo dos equipamentos, instrumentais, móveis e edificações), além dos custos fixos indiretos (higienização, segurança, energia e água). Foi também calculada a participação do Ministério da Saúde no financiamento e descritos os fatores que influenciam o comportamento dos custos.

**RESULTADOS:**

O investimento para implantação de uma Equipe de Saúde Bucal modalidade tipo I foi de R$29.864,00. Os custos operacionais de uma Equipe de Saúde Bucal modalidade tipo I situaram-se em torno de R$95.434,00 por ano. Os incentivos financeiros do Ministério da Saúde para investimentos cobriram 41,8% dos investimentos com implantação, enquanto o município participou com 59,2% do total. Para os custos operacionais, a participação do Ministério da Saúde foi de 33,1%, enquanto o município participou com 66,9%. Dentro dos custos operacionais, o elemento de maior peso foram os salários, representando 84,7%.

**CONCLUSÕES:**

Problemas com a regularidade no abastecimento dos insumos e manutenção de equipamentos influenciam sobremaneira na composição dos custos, além de reduzir a oferta de serviços à população-alvo, resultando em provável ineficiência do serviço. Sugere-se o cofinanciamento estadual, especialmente para cobrir o custo operacional da equipe.

## INTRODUCTION

The specific public federal funding for oral health care in Brazil was extended with the inclusion of oral health care teams in the Family Health Care Program, in 2000 ^a^. The Brazilian National Oral Health Policy (PNSB) has consolidated the funding to specialized and primary care, with a substantial increase from R$83,416,613.81 in 2003 to R$916,031,482.62^b^ in 2014, an increase of over 10.9 times over the period^c,d^. This process signaled an important advancement for the development of the Brazilian Unified Health System’s (SUS) principles in this field^18^. However, the increased oral health care funding in the Family Health Care Strategy over the last few years may not be enough for its maintenance^11^.

The national health care services of other countries warn about the need for economic studies to support decision making in realities of scarce resources^2,9^. However, a study based on systematic reviews on health economics in dentistry showed that from the 73 reviews selected, only 12 drew conclusions based on economic data, and two of them only focused on analyzing the dental service^16^. There is great emphasis in economically evaluating preventive programs^13,16,18^.

The only study on the topic that has recently been published in Brazil evaluated the cost of a specialized public dental service^8^ and showed that the cost with human resources accounted for over 66.0% of the total cost. A study from Cuiabá (2009) on a public dental service showed that salaries accounted for around 79.0% of the service costs^e^. Health economics has supported the optimized use of available resources for ensuring the best health care to the population, considering limited funds^1,4^. In this sense, it may contribute with subsidies that allow managers to make plans based on concrete data and the closest as possible to local realities.

So far, no studies evaluating the costs of Oral Health Care Teams in the Family Health Care Strategy were published in Brazil. Taking this gap into account, this study aimed to estimate the investments and operational costs required to implement a type I Oral Health Care Team (OHCT) in the Family Health Care Strategy.

## METHODS

This was an economic evaluation study^5,20^ that used the total absorption costing method, which represented the real operational and investment costs in a certain period when an Oral Health Care Team was implemented in the city of Salvador, BA, Northeastern Brazil in the Family Health Care Strategy (2009 to 2012). The case described refers to a type I oral health care team connected to two family health care teams providing care to 9,232 people. The Ministry of Health’s and municipality’s share in the investments and operational costs of an Oral Health Care Team was calculated in this strategy, and factors that influenced the behavior of costs were identified.

To determine the amount of investments in civil projects, the physical dimensions of the family health care unit (FHCU) and the dental office in m^2^ were considered, as they were the subject of the study. Thus, a value per m^2^ (R$1,100.00) that was practiced in Salvador was obtained for the construction of health care units via procurement processes in 2012. Based on the measurement of the total physical area in the FHCU itself and the dental office (DO), the investment in total physical area (ITPA) and the proration for the investment in the dental office area (IDOA) were obtained. Thus, ITPA = m^2^FHCU × R$m^2^ and IDOA = m^2^DO × R$m^2^ were obtained; that is, the investment amount in the physical area of the dental office is the dental office size in meters multiplied by the squared meter value.

The values of equipment, furniture, and instruments were obtained from Asi Index information system, which is the computerized and integrated system for management of materials and supplies, purchases, procurement processes, tenders, property, and suppliers that was developed by Link Data for Salvador’s Municipal Administration. Total investment was calculated by the composition of investments in civil projects and facilities, investments in equipment, furniture, and instruments. Annual rate-based depreciation, whose percentages are established by the Federal Revenue Office by the laws governing corporate income tax, was deducted from the total investment values ^f^. The depreciation rate for building was applied at 4.0% a year, considering a useful life of 50 years. Regarding equipment, a rate of 10.0% per year was applied, considering a useful life of ten years.

The depreciation calculation in the 2009-2012 time series was performed by linear retroactive accounting, and it used 2012 depreciation value as reference. Thus, a single value was adopted for years 2009, 2010, and 2011. The year of 2012 was the base for assessing the investments and operational costs in the study^6^.

Regarding the operational costs, the reference period was the 2009-2012 time series. The month of December 2012 was adopted for assessing the monetary values in effect. The time series enabled assessing the consumables, so an annual average could be found and thus variable costs could be calculated. The data sources for the amount of consumables were the filed requests of all monthly requests for supplies. Table 1 shows the values of investment components and the unit price quantities of each cost component, as well as the total cost for the year of 2012.

**Table 1:** Quantities, unit and total pricesa of each cost component (variable, direct fixed, and indirect fixed costs), including their respective data sources for funding an oral health care team in the Family Health Care Strategy in 2012. Salvador, BA, Northeastern Brazil.

Cost component (quantity, unit and total prices in R$)
Variable costs
Consumables (quantity, unit and total prices in R$)
70% Alcohol (156, R$3.03 = R$472.68), 10 L sharp container (10, R$1.46 = R$14.60), gauze pads 6.5 x 6.5 (90, R$64.00 = R$5,760.00), 3-0 chromic catgut suture thread with needle (8, R$19.68 = 157.44), adhesive autoclave tape (10, R$4.08 = R$40.80), adhesive autoclave tape (4, R$2.85 = R$11.40), disposable cap (1, R$6.17 = R$6.17), scalpel blade no. 22 (2, R$9.00 = R$18.00), scalpel blade no. 24 (0.5 , R$9.00 = R$4.50), disposable surgical glove no. 8.0 (6,5. R$160.00 = R$1,040.00), L s (100, R$10.00 = R$1,000,00), size L latex procedure glove (10, R$21,00 = R$210,00), size M latex procedure glove (100, R$10,00 = R$1,000.00), disposable surgical masks (14.5, R$8.00 = R$116.00), suture needle and thread (4, R$19.92 = R$79.68), short dental needle (3, R$10.46 = R$31.38), cotton balls (200, R$1.43 = R$286.00), dental cotton roll (50, R$0.69 = R$34.50), dental etching agent (4, R$0.94 = R$3.76), 1035 cylinder diamond drill bit (1, R$2.50), 1093 cylinder diamond drill bit (1, R$2.00), oval shofu finish drill bit (20, R$3.79 = R$75.80), calcium hydroxide cement (2, R$7.33 = R$14.66), calcium hydroxide PA (4, R$2.23 = R$8.92), powder ionomer cement (2, R$6.18 = R$12.36), powder ionomer cement (3, R$9.43 = R$28.29), liquid ionomer cement (2, R$4.19 = R$8.38), liquid ionomer cement (3, R$5.84 = R$17.52), dental cream (20, R$0.78 = R$15.6), adult toothbrush (450, R$0.25 = R$112.50), children’s toothbrush (450, R$0.22 = R$99.00), silver filings (4, R$40.18 = R$160.72), mercury for dental amalgam (6, R$40.00 = R$240.00), dental carbon paper (2, R$1.93 = R$3.86), prophylaxis paste (5, R$2.49 = R$12.45), 4mm abrasive steel strip (2, R$6.03 = R$12.06), 6MM abrasive steel strip (2, R$5.70 = R$11.40). Total: R$8,083.47/Approximate total for calculation: R$8,084.00.

Direct fixed costs
Salaries: Dental surgeon (R$64,740.48), approximate value for calculation (R$64,741.00); Oral Health Assistant (R$16,067.40), approximate value for calculation (R$16,067.00).
Maintenance contract: R$2,778.00. Depreciation calculation (10% a year).
Equipment: Automatic mechanic amalgamator (R$850.00), Autoclave - pro rata calculation per m^2^ (R$808.35), Dark Chamber (R$53.26), High speed handpiece (R$183.80), Chair (R$2,940.00), Stool (R$580.00), Microengine (R$370.00), Compressor (R$1,514.00), Contra-angle handpiece (R$210.07), Curing light (R$450.00), Stainless steel amalgam carrier (R$13.00), Ultrasonic scaler (R$1,903.00), Air conditioning (R$810.00), Mobile X-ray machine (R$3,297.00). Total: R$13,982.48.
Furniture: Dental display cabinet with wooden door (R$336.00), Fixed chair (R$99.00), Swivel chair (R$194.96), Desk with a drawer (R$205.00), Paper towel holder (R$76.00), 80-liter refrigerator (R$519.93), Sharps container holder (R$38.00). Total: R$1,468.89. Approximate value for calculation R$1,469.00.
Instruments: Right-angled root elevator (R$7.83), Left-angled root elevator (R$7.15), Curved straight elevator no. 302 (R$7.19), Curved straight elevator no. 303 (R$7.19), Pediatric straight elevator (R$7.19), Straight elevator no. 301 (R$7.19), Dycal applicator (R$5.10), Burnisher (R$3.50), Rectangular stainless steel tray size 30x40 cm (R$38.00), Rectangular stainless steel tray size 30x25 cm (R$46.20), Scalpel handle no. 03 (R$3.95), Surgical box with lid size 0.20x10x0.5 cm (R$26.45), Surgical box with lid 0.32x0,16x0.8 cm (R$81.72), Straight chisel (R$12.00), Amalgam condenser no. 01 (R$4.21), Amalgam condenser no. 02 (R$4.22), Double ended alveolar curette no. 86 (R$4.21), Gracey surgical curette no. 11/12 (R$4.21), Gracey surgical curette no. 13/14 (R$4.21), Gracey surgical curette no. 9/10 (R$4.21), Dentin excavator no. 14 (R$4.21), Periodontal curette no. 13/14 (R$4.21), Periodontal curette no. 5/6 (R$4.21), Periodontal curette no. 7/9 (R$4.21), Periosteal elevator (R$5.25), Hoe scaler no. 4/8 (R$5.23), Frahm carver (R$3.22), Hollenback carver (R$3.22), Discoid/cleoid carver (R$3.22), Spatula no. 7 (R$4.56), small Heidemann spatula (R$4.56), Dental spatula (R$4.56), Dental mirror with handle (R$1.98), Explorer probe no. 5 (R$2.83), Posterior periodontal scaler (R$4.21), Adult forceps no. 150 (R$19.76), Adult forceps no. 151 (R$19.59), Adult forceps no. 16 (R$19.76), Adult forceps no. 17 (R$19.59), Adult forceps no. 18L (R$21.58), Adult forceps no. 18R (R$21.58), Adult forceps no. 44 (R$21.84), Adult forceps no. 65 (R$19.76), Adult forceps no. 68 (R$19.59), Adult forceps no. 69 (R$19.59), Pediatric forceps no. 1 (R$18.00), Pediatric forceps no. 4 (R$18.00), Pediatric forceps no. 5 (R$18.00), Pediatric forceps no. 6 (R$18.00), Pediatric forceps no. 18D (R$18.00), Forceps no. 101 (R$19.63), Forceps no. 203 (R$19.59), Forceps no. 21 (R$19.59), Forceps no. 210 (R$19.59), Forceps no. 23 (R$19.59), Mortar (R$7.50), Bone file (R$8.85), Clinical tweezers (R$2.86), Curved gouge forceps (R$2.86), Straight gouge forceps (R$32.94), Tweezers for utensils (R$31.94), Pestle (R$6.50), Needle holder (R$13.00), Stainless steel amalgam holder (R$13.00), Matrix retainer (R$14.32), Clamp forceps (R$5.23), Aspirating syringe (R$17.84), Periosteal elevator (R$8.76), 12x12 cm steel lid (R$26.60), Metal-cutting scissors (R$17.80), Curve scissors (R$15.25). Total: R$4,183.36.

Depreciation calculation (4% a year in civil projects): Dental office area (9.3 m^2^)^b^ (R$10,230.00), Gross floor area (304.88 m^2^)^c^. Total: R$409.20/Grand Depreciation Total: R$2,372.76/Approximate total for calculation: R$2,373.00.

Indirect fixed costs (pro rata calculation per m2)
Water and electricity (R$488.00). Human resources: Cleaning 1 and 2 (R$467.00) and security (R$348.00). Cleaning and conservation supplies (R$88.00).

^a^ In 2012, U$S1 = R$1.96.

^b^ Procurement process document dated 2012 for the health care unit construction. Measurement at the family health care unit itself. We prepared it ourselves based on data obtained from Asi Index system, except for civil projects (value per m^2^ × dental office size in meters). Value per m^2^ = R$1,100.00.

^c^ The value was not part of the composition of investments in the oral health care team.

To compose the structure of operational costs, we considered the direct costs, which included variable costs (consumables), direct fixed costs (salaries, costs with maintenance companies, fixed capital depreciation of instruments, furniture, and facilities); and the indirect fixed costs (utilities, cleaning, and cleaning and security consumables), which were accounted on a pro rata basis considering the square meter value of the dental office. To determine cost, the cost object was defined; i.e., the element whose cost we wished to know. In this case, we worked with the total cost of an oral health care team, which corresponds to the sum of direct and indirect fixed costs. Direct costs are subdivided in fixed and variable costs^6^.

The OHCT operates 40 hours a week by performing individual clinical actions in seven shifts and by performing collective actions and making house and planning visits in three shifts, as recommended by the Ministry of Health. It distributes treatments as recommended, considering a 30-day month, of which 22 are working days. Thus 15 days of individual clinical care must be offered, which would correspond to scheduling 360 patients a month for appointments and to having seven days of educational collective and planning activities.

The collective actions refer to activities of supervised brushing with topical use of fluorine in day care and elementary schools in the area. The production of procedures was identified based on the Outpatient Production Record of SUS’ Outpatient Information System^g^.

To measure the total individual care provision, this team’s schedule of appointments from 2009 to 2012 was analyzed. The individual appointments were classified as: a) scheduled, b) provided, c) urgent, and d) rescheduled or not provided. The total of no-show patients was also recorded. This analysis was possible because the same oral health orderly was maintained during the studied period.

To measure the executed procedures, the 2009-2012 time series regarding this team’s outpatient production (which is recorded by SUS’ Outpatient Information System) was obtained in the very health care unit and analyzed. The Ministry of Health informs its participation in investments and operational costs by these main ministerial directives: 2489/2008^h^, 2372/2009^i^, 1599/2011^j^, and 366/2012^k^.

## RESULTS

The investment to implement a type I oral health care team was R$29,864.00. The annual operational costs for the type I team stayed around R$95,434.00 for year 2012. The analyzed FHCU’s oral health care team treated 1,324 patients, totaling 3 procedures. The Ministry of Health cooperated with R$44,079.00 to implement and fund the type I oral health care team.

Investment in physical areas was R$10,230.00. Investment in equipment totaled R$13,982.00; in furniture, R$1,469.00; and in instruments, R$4,183.00. The total amount of investment components was R$29,864.00 for obtaining a physical structure of equipment, furniture, and instruments. Investment in civil projects only corresponds to the physical area of the dental office.

The annual human resource costs, which are directly responsible for the outpatient production, were always above 70.0% of the total costs, and were observed to rise yearly, albeit with a certain stability, as they are fixed costs (Table 2).

**Table 2:** Operational costsa and total cost of operation of the evaluated type I oral health care team in the Health District of Liberdade between 2009 and 2012. Salvador, BA, Northeastern Brazil.

Cost type	Values per year (in *reais*)			

	2009	2010	2011	2012
Variable costs				
Consumables	5,447	7,981	3,930	8,084
Direct fixed costs				
Salaries				
Dental surgeon	56,952	60,684	62,464	64,741
Oral health care assistant	14,714	15,690	15,886	16,067
Equipment maintenance	2,418	2,418	2,778	2,778
Depreciation calculation^b^	2,057	2,057	2,057	2,373
Indirect fixed costs				
Water and electricity (pro rata calculation)	412	434	459	488
Supporting staff (cleaning calculated pro rata)	350	410	409	467
Supporting staff (security calculated pro rata)	308	320	334	348
Cleaning and conservation supplies (pro rata calculation)	53	79	58	88

Total	82,711	90,073	88,375	95,434

^a^ In 2009, U$S1 = R$2; in 2010, U$S1 = R$1.76; in 2011, U$S1 = R$1.68; in 2012, U$S1 = R$1.96.

^b^ For years 2009, 2010, and 2011, the depreciation value was considered via a linear method, based on investments in 2009. For 2012, the new assets that were designed for a new unit were considered.

The monetary values of annual maintenance costs within 2009 and 2012 pointed towards stability for the years of 2009 and 2010, with an increase in 2011, which remained stable in 2012 (Table 2). Maintenance was suspended during that time due to lack of payment to the company in charge. This reduced the use of the service and changed the costs behavior. Variable costs were reduced and fixed costs increased proportionally when the team production was the lowest (less procedures and individual appointments). There was no maintenance in the months of June, July, and August 2011 and 2012, for example. This caused rescheduled and canceled appointments for many patients, which led to a lack of services offered, as most team costs remained, especially the direct fixed costs. In 2012, the average unit cost per procedure was approximately R$25.07, in an analysis that did not distinguished categories among the procedures for calculating the unit value. The procedures that were performed in smaller numbers were found to have higher proportional costs (Table 3).

**Table 3:** Structure of operational costsa regarding the base year of 2012 of the evaluated type I oral health care team in the Health District of Liberdade. Salvador, BA, Northeastern Brazil.

Categories	Annual cost in *reais*	Average unit cost^a^ (R$/unit)	Vertical analysis^b^ (% on the total cost)
Direct costs (A)			
Variable costs	8,084	2.12	8.47
Consumables			
Total (1)	8,084	2.12	8.47
Direct fixed costs			
Salaries (total)	80,808	21.23	84.67
Dental surgeon salary	64,741	17.01	67.84
Oral health care assistant salary	16,067	4.22	16.83
Dental office maintenance (total)	2,778	0.73	2.91
Depreciation calculation (total)	2,373	0.62	2.49
Civil projects	409	0.10	0.43
Equipment	1,398	0.36	1.47
Furniture	147	0.03	0.15
Instruments	418	0.10	0.44
Total (2)	85,959	22.58	90.07
Total direct costs (A = 1 + 2)	94,043	24.70	98.54
Indirect fixed costs (support) (B)			
Electricity/Water (pro rata calculation)	488	0.13	0.51
Human resources (cleaning calculated pro rata)	467	0.12	0.49
Cleaning and conservation supplies (pro rata calculation)	88	0.02	0.09
Human resources (security calculated pro rata)	348	0.09	0.36
Total (B)	1,391	0.36	1.46
Total cost (+ B)	95,434	25.07	100
Number of procedures/year	3,561	-	-

^a^ In 2009, U$S1 = R$2; in 2010, U$S1 = R$1.76; in 2011, U$S1 = R$1.68; in 2012, U$S1 = R$1.96.

^b^ Unit cost = total cost, divided by the number of procedures conducted/year.

^c^ Vertical analysis (%): relative contribution of each cost item in relation to the total cost. Equation: Vertical analysis = Cost element/total cost x 100.

Maintenance was found to have an annual cost of R$2,778.00, with a unit cost of R$0.73 and a vertical analysis of 2.9%. Despite the maintenance cost having a small average unit value, not having it led to a considerable loss of productivity and use of the service. Total direct cost corresponded to R$94,043.00 and represented a unit cost of R$24.70, with vertical analysis of 98.5% in 2012 (Table 3).

Within the evaluated period, these fixed costs were observed to have a higher contribution. They are composed of human resource salaries directly associated with production, due to the small contribution from variable costs, such as the one with consumables. The low percentage contribution from consumables was a result from lower productivity.

Lack of regularity in input provision may also have influenced variable cost behavior. In 2009, the rate of rescheduled and untreated patients was the lowest in the period (6.9%). In 2011, in turn, that rate was 52.0% (Table 4). The probable reasons might be related to lack of input stock replenishment, besides problems in equipment maintenance, as already mentioned. The dental surgeon seems to have chosen to perform over three procedures per treatment, to keep productivity from being too low (Table 4).

**Table 4:** Number of annual procedures executed by the evaluated type I oral health care team in the Health District of Liberdade between 2009 and 2012. Salvador, BA, Northeastern Brazil.

Type	Procedures per year			

	2009	2010	2011	2012
Total individual appointments	1,787	1,399	850	1,324
Scheduled	2,320	2,315	2,322	2,477
Treated	1,513	1,344	746	1,184
Emergencies	274	55	104	140
Rescheduled and untreated	160	172	1,213	663
No-shows	647	799	363	630
Total procedures conducted	3,230	4,293	3,493	3,806
Health promotion and prevention initiatives	-	493	102	108
Individual clinical procedures	2,982	3,527	3,274	3,370
Surgical procedures	198	197	117	161
Emergency procedures	50	76	-	76

Source: Prepared by the authors. The appointment data are based on the unit’s appointment schedule and on its outpatient production, on data from the Outpatient Information System.

There was an increase observed in the number of procedures between 2009 and 2010 (3,230 and 4,293 procedures, respectively), followed by the rise in variable costs. From 2010 to 2011, we observed a reduction in the number of procedures (4,293 and 3,493, respectively) as well as in the variable costs. Between 2011 and 2012 procedures were observed to increase in number, thus also raising variable costs. In 2012, the increase corresponded to R$4,154.00 in an absolute value, a difference originated from the receipt of consumables that had not been received in 2011. Most years were identified to have urgent treatments scheduled that were not filed in the information system. Restorative procedures were found to outnumber all others every year, and they were followed by surgical procedures. Health promotion and prevention initiatives were not recorded in 2009; in the remaining years, small numbers were observed, which indicates lack of change in this health care unit’s model, with little emphasis on health surveillance.

The percentage share of the Ministry of Health and the municipality in the investment corresponded to 41.8 and 59.2%, respectively, which represents significant contribution from the municipal level. Operational costs were found to have a ministerial contribution of 33.1%; the municipality contribution, in turn, accounted for 66.9%, which represented a high percentage for the municipal level, with a good one from the Ministry of Health. From the total amount of funds used for both investments and operational costs, 35.2% were supported by the Ministry of Health, and the municipality contributed with a 64.8% share, which shows an even higher participation from the municipality in the operational costs (Table 5).

**Table 5:** The municipality’s and the Ministry of Health’s share in reais* and % in the total expenditures with an oral health care team in the Family Health Care Strategy, 2012.

Expenditure type	Investment	% of investment	Cost	% of cost	Total	Percentage share
Financial incentives from the Ministry of Health	12,469	41.8	31,610	33.1	44,079	35.2
Municipality’s investment and operational costs	17,395	59.2	63,824	66.9	81,219	64.8
Total expenditures from an oral health care team in 2012	29,864	100	95,434	100	125,298	100

* In 2012, U$S1 = R$1.96

## DISCUSSION

The investment to implement a type I OHCT was R$29,864.00, whereas the annual operational costs it required in 2012 were R$95,434.00, thus totaling R$125,298.00. In this sense, the Ministry of Health’s financial incentive towards the implementation covered as much as around half of the amounts with capital costs. Regarding the operational costs, the Ministry of Health contributed with 35.2% of the total, whereas the municipality, with 64.8%.

Regarding investments, the study showed that capital costs corresponded to 23.8% of the total cost. Ferreira and Loureiro^8^ (2008) also showed, albeit under a distinct methodological procedure, that investments (capital cost) towards an oral health specialty center amounted to 38.3% of the total, with a unit dental office value of R$47,098.00. A study with a similar methodology to the study by Ferreira e Loureiro^8^ found that the investment in capital costs corresponded to 40.5% for a specialized dental service in Cuiaba, with an unit dental office cost of R$52,458.00^d^.

The lower capital cost found in this study may be related to the pro rata distribution of the cost with physical projects, as OHCT shares a physical space with the remaining Family Health Care Strategy services. Besides that, Asi Index system of Salvador’s Municipal Health Care Office may also always have lower prices under the smallest price logic in public procurement processes. In this sense, the studies reinforce the need to include capital cost analysis to calculate the total cost, and this capital cost is essential^7,8,10^.

This study also showed that direct operational costs with human resources were found to account for the greatest share of the total cost (84.7%) and variable costs were observed to account for 8.5% in 2012, as also found by other authors^7,10,15^. Human resource costs have represented from 66.4% to 79.4% of the total cost^10^. Variable costs (consumables), in turn, have been from 9.8% to 10.3%^7^. A study by Rosa and Canduro Neto^15^ showed that human resources costs accounted for 88.0% of the total cost; however, the authors had not included capital cost and treatments were restricted to children.

In this sense, despite the methodological differences, the historic time and the specific reality of these public dental services indicate that direct fixed costs with staff (salaried) correspond to the greatest part of the total cost, and are always above 70.0% of the total operational costs. Salary variations may also be an important differentiating factor.

Concern with service costs and payment methods has been emphasized^19,24^. However, none of these studies analyzed time series of the service cost, which prevented us from characterizing the factors that influence cost. This study, besides identifying these factors, also showed that the available human resources were underused due to infrastructure problems, which resulted in low use of this public service. That is, fixed costs were observed to remain high without producing a better use of the service. An investigation identified a relationship between the characteristics of the local government, the organization of dental services, and the implementation of Brazilian National Oral Health Policy in Salvador, where the lack of financial autonomy from its Municipal Health Care Office at that time led to problems with supply and regularity of inputs in the network, as well as with maintenance of dental equipment^17^. Integrating economic principles in management processes is essential, as managers’ decisions must include an analysis of the complexity of health care management processes, hence recognizing management as the political and administrative conduction of a system^2^.

Another study in Salvador found management problems in the municipal administration, where the health care officer’s little autonomy to manage funds was highlighted, with an instability in providing health care units with basic supplies, such as consumables^23^. That hindered the sustainability of oral health care initiatives, as found in this study, as dental work processes depend on material technologies, equipment, and inputs.

As of 2006, due to the already mentioned ministerial directives and pursuant to *Pacto pela Saúde* [Pact for Health], the way to transfer funds changed, with five funding blocks having been created. Brazilian National Oral Health Care Policy has funds ensured in three blocks, in basic health care; in medium and high complexity; and in management. In this sense, as of 2006’s Pact, the oral health care teams that operate in the Family Health Care Strategy are funded by the basic health care block by fixed Basic Health Care Floor and incentives from variable Basic Health Care Floor.

To Kornis et al.^11^, the innovations brought about by the Management Pact may generate increased autonomy to local managers when using funds. Salvador’s oral health care benefited from these innovations, as part of the oral health care teams joined the Program for Improved Access and Quality in October 2011, including OHCT into its analysis. Thus, this federal funding line was received by the 2009-2012 management board in 2012. However, this incentive yielded no direct benefits to OHCT.

The Health Department of the State of Bahia transfers funds to the Family Health Care Strategy, but it establishes no specific funding for oral health care teams. The municipal level is responsible for the specific costs remaining after the transference of funds from the federal government. Such transference to states and municipalities is made on a regular basis straight from Brazilian National Health Care Fund, regardless of agreements or similar instruments. Moimaz et al.^14^ confirm that specific oral health care funding is restricted to the federal level. This study also showed that the financial incentives for supporting PNSB are not enough if they do not include investments from other federation agents, as prescribed by Constitutional Amendment 29^21^.

This study results reinforce the need for a federative pact in the co-financing and consolidation of stable fund sources for the health care sector^11,22^, which should include public dental care. These studies also showed that the increased federal funding for primary health care and strategic programs has been important, but insufficient^12,21^. The investment to implement a type I oral health care team corresponds to less than 25.0% of the total cost, and it must be considered in economic studies, as this component may change results. Thus, it is relevant to take it into account, as deterioration of equipment and instruments, which are investment items, may lead to operational inefficiency.

This team’s cost analysis in this study also considered the costs in the oral health care team’s three shifts which are theoretically dedicated to house visits, planning, team meetings, and collective initiatives. Economic studies that focus on different care models must be fostered, as the studies are mainly focused on preventive programs^16,18^. Conducting studies is also recommended for estimating costs associated with different scenarios in the Brazilian reality, such as oral health care teams with Oral Health Care Technicians (type II), teams operating under the traditional health care model, and also as an economic analysis of collective initiatives only conducted by oral health care technicians and assistants, rather than by third level professionals.

The main gap in this study was evaluating a single case without comparing services from distinct realities or comparatively analyzing their efficiency. Besides that, cost-benefit or cost-effectiveness was not analyzed, and these are common procedures when evaluating preventive programs^3,13^. Studies for analyzing efficiency, cost-benefit, and cost-effectiveness are therefore indicated. Despite that, this study contributed in the analysis of cost behavior, by indicating that problems in the replenishment of dental consumables and in the continuity of equipment maintenance greatly jeopardize the use of this service^25^. Upon considering co-financing for oral health in the Family Health Care Strategy, the contributions from federal and municipal governments are identified. Thus, Bahia state, as well as other states who still do not do it, are suggested to contribute with specific funds regarding the operational costs for oral health care teams or public dental services in their territories.

## Footnotes

a Ministério da Saúde. Portaria nº 1.444, de 28 de dezembro de 2000. Estabelece incentivo financeiro para a reorganização da atenção à saúde bucal prestada nos municípios por meio do Programa de Saúde da Família. *Diario Oficial Uniao*. 29 dez 2000.

b In American Dollar, U$27.171.535,0 in 2003 and US$389.800.631,0 in 2014.

c Ministério da Saúde, Fundo Nacional de Saúde. Portal da Transparência. Brasília (DF); s.d. [cited 2015 Jul]. Available from: http://www.portaldatransparencia.gov.br

d In 2009, U$S1 = R$2; in 2010, U$S1 = R$1.76; in 2011, U$S1 = R$1.68; in 2012, U$S1 = R$1.96.

e Simões CAD. Avaliação econômica de um serviço público de saúde bucal de Cuiabá-MT [dissertation]. Campinas (SP): Centro de Pesquisas Odontológicas São Leopoldo Mandic; 2009.

f Ministério da Fazenda, Secretaria da Receita Federal do Brasil. Depreciação de bens do ativo imobilizado. Brasília (DF); s.d. [cited 2012 Jul]. Available from: http://www.receita.fazenda.gov.br/pessoajuridica/dipj/2001/PergResp2001/pr353a366.htm

g Ministério da Saúde, DataSus, Informações de Saúde (TABNET). Assistência à saúde. Produção ambulatorial (SIA/SUS). Brasília (DF): Ministério da Saúde [cited 2013 May 5]. Available from: http://www2.datasus.gov.br/DATASUS/index.php?area=0202&id=19122

h Ministério da Saúde. Portaria nº 2.489, de 21 de outubro de 2008. Define valores de financiamento do Piso da Atenção Básica Variável para a estratégia de Saúde da Família e de Saúde Bucal, instituídos pela Política Nacional de Atenção Básica. *Diario Oficial Uniao*. 22 out 2008.

i Ministério da Saúde. Portaria nº 2.372, de 7 de outubro de 2009. Cria o plano de fornecimento de equipamentos odontológicos para as Equipes de Saúde Bucal na Estratégia Saúde da Família. *Diario Oficial Uniao*. 9 out 2009.

j Ministério da Saúde. Portaria nº 1.599, de 9 de julho de 2011. Define valores de financiamento do Piso da Atenção Básica Variável para as Equipes de Saúde da Família, Equipes de Saúde Bucal e aos Agentes Comunitários de Saúde, instituídos pela Política Nacional de Atenção Básica. *Diario Oficial Uniao*. 11 jul 2011; Seção 1:50.

k Ministério da Saúde. Portaria nº 366, de 5 de março de 2012. Define os recursos financeiros para aquisição de equipamentos odontológicos destinados aos Municípios que implantaram Equipes de Saúde Bucal na Estratégia Saúde da Família. *Diario Oficial Uniao*. 6 mar 2012; Seção 1:91.
